# ETISTP: An Enhanced Model for Brain Tumor Identification and Survival Time Prediction

**DOI:** 10.3390/diagnostics13081456

**Published:** 2023-04-18

**Authors:** Shah Hussain, Shahab Haider, Sarmad Maqsood, Robertas Damaševičius, Rytis Maskeliūnas, Muzammil Khan

**Affiliations:** 1 Department of Computer Science, City University of Science and Information Technology, Peshawar 25000, Pakistan; shahhussainf@gmail.com; 2 Faculty of Informatics, Kaunas University of Technology, 51368 Kaunas, Lithuaniarytis.maskeliunas@ktu.lt (R.M.); 3 Department of Applied Informatics, Vytautas Magnus University, 44404 Kaunas, Lithuania; 4 Faculty of Applied Mathematics, Silesian University of Technology, 44-100 Gliwice, Poland; 5 Department of Computer & Software Technology, University of Swat, Swat 19200, Pakistan; muzammilkhan86@gmail.com

**Keywords:** brain tumor identification, brain tumor classification, medical image processing, image segmentation, deep learning, survival prediction

## Abstract

Technology-assisted diagnosis is increasingly important in healthcare systems. Brain tumors are a leading cause of death worldwide, and treatment plans rely heavily on accurate survival predictions. Gliomas, a type of brain tumor, have particularly high mortality rates and can be further classified as low- or high-grade, making survival prediction challenging. Existing literature provides several survival prediction models that use different parameters, such as patient age, gross total resection status, tumor size, or tumor grade. However, accuracy is often lacking in these models. The use of tumor volume instead of size may improve the accuracy of survival prediction. In response to this need, we propose a novel model, the enhanced brain tumor identification and survival time prediction (ETISTP), which computes tumor volume, classifies it into low- or high-grade glioma, and predicts survival time with greater accuracy. The ETISTP model integrates four parameters: patient age, survival days, gross total resection (GTR) status, and tumor volume. Notably, ETISTP is the first model to employ tumor volume for prediction. Furthermore, our model minimizes the computation time by allowing for parallel execution of tumor volume computation and classification. The simulation results demonstrate that ETISTP outperforms prominent survival prediction models.

## 1. Introduction

Tumor refers to undesired tissues, which may result in being malign or benign. Brain tumors are a complex and heterogeneous group of diseases. There are various types of brain tumors, i.e., gliomas, meningiomas, and pituitary [1,2,3,4]. Gliomas are the most common types of brain tumors, accounting for approximately 80% of all malignant brain tumors. Gliomas rapidly spread to other tissues, they are hard to detect, and they have the highest death ratio among the aforementioned tumor types [5,6]. Gliomas are further classified into low-grade glioma (LGG) and high-grade glioma (HGG) based on their cellular and genetic characteristics. LGGs are less aggressive and have better prognoses than HGGs. Contrarily, HGG occurs in the spinal cord and brain; it exhibits a faster growth rate, which makes it highly dangerous and hard to treat. Hence, HGGs are more malignant and have a higher mortality rate.

Diagnosing and treating brain tumors are challenging tasks for healthcare systems globally, and remain dependent on a patient’s survival chances. The current standard of care for brain tumor patients includes surgery, chemotherapy, and radiotherapy. However, the effectiveness and side effects depend on several factors, such as tumor grade, size, location, patient’s age, and overall health. Brain tumors are one of the biggest contributors to deaths in human beings worldwide [7,8,9]. Once a brain tumor is diagnosed, a patient’s survival rate increases by 36% for the next five years, thereby demanding the urgent attention of the research community [10,11].

Since tumor diagnosis requires careful assessment to extend a patient’s survival chances, clinical diagnosis assisted by technology has gained immense importance in healthcare systems [12,13,14], giving rise to technology-assisted diagnoses in healthcare systems [15]. Moreover, the threat level of a tumor impacts the survival time, which depends on various parameters, such as the patient’s age, tumor type, tumor stage, etc. [16,17]. This demands the efficient classification of tumors in terms of severity level, which is referred to as the tumor grade [18].

To address this issue, the existing body of knowledge [19] provides several models. Survival prediction models have been developed to aid in the diagnosis and treatment of brain tumors. These models use different clinical parameters, such as patient age, tumor size, tumor grade, and gross total resection (GTR) status, to predict patient survival time. The authors in [20] present a novel model for brain tumor identification and patient survival prediction. Similarly, the authors of [21] predicted tumor growth using parameter population distribution (PPD). Furthermore, the authors in [22] employed multi-parametric magnetic resonance images (mpMRI) to identify tumors and analyze their progression; they are used to predict survival time based on gross total resection (GTR) and patient age. However, existing models lack the integration of the aforementioned parameters for identification, classification, and survival prediction. Moreover, these models consider two-dimensional (2D) tumor sizes, which degrade their performance. Furthermore, existing models have shown limitations in accuracy due to the complexity and heterogeneity of brain tumors.

One potential solution to improve the accuracy of survival prediction models is to integrate multiple clinical parameters, including tumor volume, into the model. Tumor volume is a critical factor in predicting brain tumor progression and patient survival, and its integration into the prediction model could improve the accuracy of survival predictions. Moreover, the parallel execution of tumor volume computation and classification could minimize the computation time, making the model more practical and efficient.

Motivated by the need for more accurate and efficient survival prediction models for brain tumors, this paper proposes a novel model, namely, enhanced brain tumor identification and survival time prediction (ETISTP), with the following contributions.

1. ETISTP enables the improved classification of gliomas with respect to different grades.
2. To the best of the authors’ knowledge, this work pioneers the use of tumor volume for survival time prediction.
3. This work integrates four different factors to enhance the accuracy of survival time prediction.
4. The proposed model reduces the computation time by enabling the parallel execution of tumor volume computation and classification.

The rest of the article is organized as follows. Section 2 critically reviews the related work on brain tumor classification and survival prediction. Section 3 details our proposed model. Section 4 evaluates the performance of the proposed method in comparison with the state-of-the-art. Finally, Section 5 includes the conclusion with future research directions.

## 2. Related Work

Brain tumors are one of the largest contributors to death in human beings worldwide, due to their severe adverse effects on the nervous system, which result in high death rates [17]. Magnetic resonance images (MRIs) are widely utilized in healthcare systems for tumor diagnosis [18]. However, manual diagnosis by medical practitioners utilizing these images remains challenging. As a result, researchers have proposed various models that significantly reduce human intervention in the diagnostic process and improve accuracy. This section provides a comprehensive analysis of the existing models by highlighting their advantages and disadvantages.

The authors in [12] fused computed tomography (CT) and MRIs to acquire new synthetic images with enriched information that could be exploited to enable enhanced diagnosis. Image segmentation enables enhanced identification of tumors. To this end, the authors in [23] presented a novel model based on generative adversarial net (GAN). Similarly, a convolutional neural network (CNN)-based model, proposed in [24], enables normalized segmentation that can be used to identify tumor regions. The authors in [25] reviewed supervised and unsupervised deep learning techniques for tumor identification. Tumor growth was analyzed in [22] using various machine learning algorithms that segment mpMRI images.

The process of successful identification initiates the classification of tumors into different types or grades. The authors of [26] classified brain tumors into three categories using a probabilistic neural network (PNN). This model extracts features using a gray-level co-occurrence matrix (GLCM), while segmentation is performed using the K-means algorithm. In addition, the authors of [21] used a classical mathematical parameter population distribution model to predict tumor growth by estimating an independent parameter vector of an independent growth curve. In [27], the authors integrated different classifiers, i.e., extra trees, random forest, and logistic regression, and achieved better prediction performance by utilizing the combined results obtained from the aforementioned classifiers. The authors in [7] presented an intelligent technique for classification. Another model proposed in [4] used fuzzy brain-storm optimization, which classifies brain tumors into gliomas, meningiomas, and pituitary categories. Similarly, MRIs were augmented through pair-wise GAN in [28]. Shubham et al. [2] presented an enhanced model based on CNN and support vector machine (SVM) to classify brain tumors into three categories, i.e., glioma, meningioma, and pituitary. Gliomas are categorized as low-grade glioma (LGG) and high-grade glioma (HGG).

The model proposed in [29] classifies gliomas into the aforementioned grades. Rajnikanth et al. [30] developed a computer-aided diagnosis and detection (CADD) system that uses convolutional neural network (CNN)-supported segmentation and classification to identify glioblastoma/glioma-class brain tumors in 2D MRI slices. The effectiveness of the CADD system was confirmed through an investigation using a benchmark and real clinical brain MRI slices, and the performances of well-known classifiers were compared. They found that SVM-Cubic achieved the highest accuracy (>98%). These results demonstrate that using CNN-assisted segmentation and classification can improve disease detection accuracy. Bajdie et al. [31] used the AlexNet convolutional neural network (CNN) to detect and classify brain tumors in magnetic resonance (MR) images, achieving an overall accuracy of 99.62%. Kurdi et al. [32] used the Harris Hawks optimization CNN (HHOCNN) in their study to enhance the accuracy of brain tumor recognition in MRI images. Firstly, they pre-processed the MR images and removed noisy pixels to decrease the rate of false tumor recognition. Then, they employed a candidate region method to identify the tumor region by examining the boundary regions using line segments, which helped to retain hidden edge details. After segmenting the region, various features were extracted and classified using a CNN model that computed the precise location of the tumor with fault tolerance. By using the nature-inspired Harris Hawks optimization algorithm, the researchers minimized the misclassification error rate and improved the overall tumor recognition accuracy to 98% on the Kaggle dataset.

The Cox proportional hazards (CoxPH) regression model is a popular prediction model that employs different parameters [33]. The authors of [34] presented an extended CoxPH model applied to the medical records of breast cancer patients. Similarly, the authors of [20,22] proposed prediction models, which took the age and GTR as inputs to estimate the survival time of a patient. These models categorize survival into three classes, i.e., long-survival, mid-survivors, and short-survivors. The tumor size and other clinical records are widely used for a patient’s survival prediction [35]. Additionally, the authors of [36,37] proposed models to estimate risk factors based on historical and current data. Moreover, the authors in [38] employed a deep learning method to diagnose Alzheimer’s disease. Moreover, SAVAE-COX [39], PubMed [40], and Page-Net [41] employ deep learning for survival time prediction. Similarly, a statistical model (SM) was proposed in [42], which efficiently predicts the survival time. SAVAE-COX takes into account transfer learning, which results in a C-Index of 0.71, making it superior to PubMed, Page-Net, and SM.

From the literature review, it is apparent that various parameters are used to estimate a patient’s survival time, including age, survival days, gross total resection status, tumor size, or tumor grade. However, the existing models rely on the use of a single parameter or a combination of a few parameters. The integration of all these parameters is still lacking in the literature, which can enhance accuracy. Moreover, the consideration of the 2D tumor size for prediction also reduces the efficiency of these models. To address these issues, this study presents a novel model that is detailed in the following section.

## 3. The Proposed ETISTP Model

This section introduces our proposed enhanced brain tumor identification and survival time prediction (ETISTP) model. ETISTP comprises five phases: pre-processing, tumor identification, tumor volume computation, tumor grade classification, and survival time prediction, as shown in Algorithm 1. The model improves brain tumor identification and classification, and predicts the patient’s survival time in terms of days. There are several types of brain tumors, including gliomas, meningiomas, and pituitary tumors [2,3,4]. The ETISTP model focuses on gliomas, which have the highest death rate worldwide [2,6].
**Algorithm 1** Pseudocode of the proposed ETISTP model.**Step 1.** Start.**Step 2.** Input Brats2020 datasets.**Step 3.** Apply pre-processing.**Step 4.** Brain tumor Identification from 3D-MRI using U-Net model, as shown in Figure 4**Step 5.** Compute tumor volume using Equation (1).**Step 6.** Classify the tumor grade based on HGG and LGG using 3D-CNN.**Step 7.** Four-factor integration to calculate the hazard value by Equation (2).**Step 8.** Using the CoxPH model to calculate the survival rate using Equation (3).**Step 11.** End.

**Figure 1:**
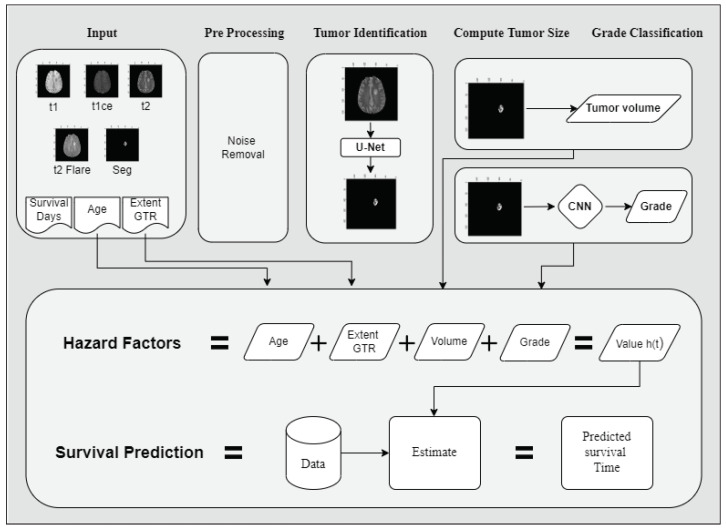
Procedural flowchart of the proposed ETISTP model.

The first phase involves pre-processing the magnetic resonance images (MRIs) to remove noise from the input images. The pre-processed MRIs are then used as input in the second phase for tumor identification through segmentation. Upon successful tumor identification, we compute the tumor volume and perform classification in terms of low-grade glioma (LGG) and high-grade glioma (HGG). The segmented image generated by the tumor identification phase serves as input for both the tumor volume computation and tumor grade classification phases. This enables the ETISTP model to carry out tumor volume computation and classification in parallel, significantly minimizing the overall response time of our proposed model. Finally, the ETISTP model predicts the patient’s survival time in terms of days based on four different parameters, namely, tumor type, tumor volume, patient’s age, and EoGTR. Figure 1 illustrates the procedural flowchart of our proposed model.

### 3.1. Pre-Processing

**Figure 2:**
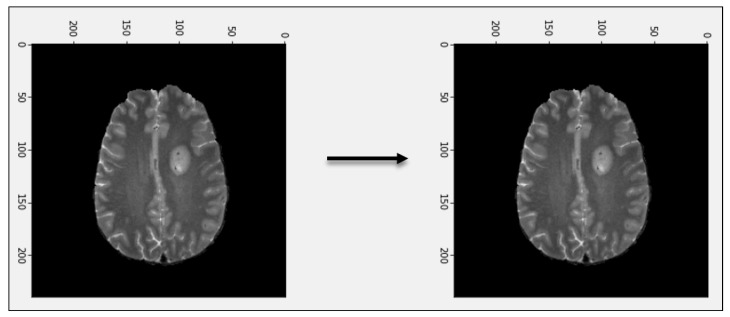
Pre-processing of the input images using a median filter.

The proposed ETISTP model begins with pre-processing of a three-dimensional (3D) source image bearing *P*, *Q*, and *R* dimensions, where *P* = {1, 2, 3, …, *p*}, *Q* = {1, 2, 3, …, *q*}, and *R* = {1, 2, 3, …, *r*}. Pre-processing eradicates noise from an input source image. To this end, we employ the median filter, which is one of the most efficient choices [43], as shown in Figure 2.

**Figure 3:**
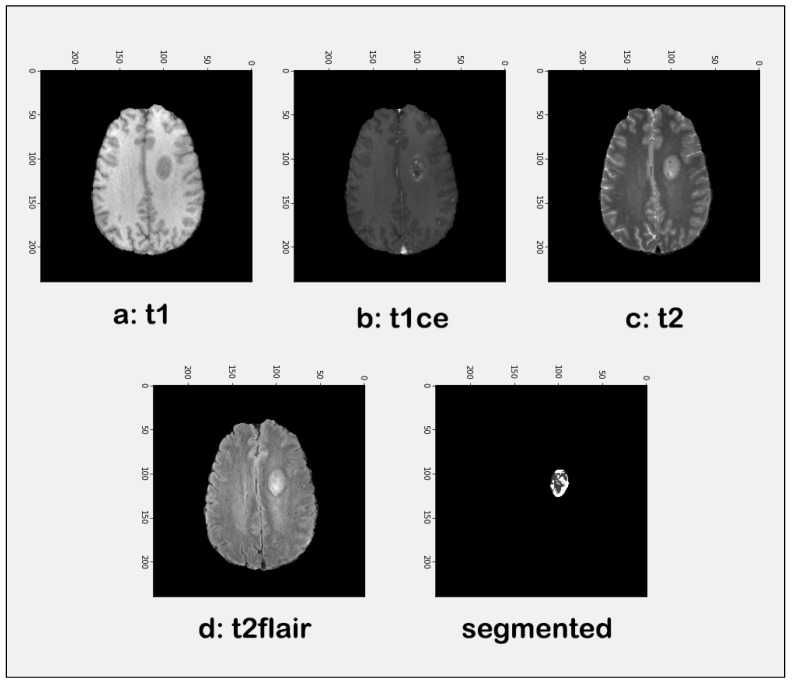
Different modalities of the images in the Brats-2020 dataset.

The ETISTP model is validated on the benchmark Brats-2020 dataset, which is publicly available on The Cancer Imaging Archive (TCIA) [10]; it comprises 3D MR images with 240 × 240 × 155 resolutions [22,44,45]. This dataset contains the MRIs of 369 patients represented in 5 different modalities, i.e., t1, t1ce, t2, t2flair, and segmented, as shown in Figure 3. The integration of data from various modalities is important for achieving higher classification accuracy [46,47]. A t1 modality refers to a 3D-weighted image with axial 2D or sagittal native images with 1–6 millimeter (mm) slice thicknesses. This modality represents the image components as black holes. Moreover, the t1ce modality is a 3D-weighted contrast-enhanced (Gadolinium) image, with an isotropic voxel size of 1 mm, showing edges of the blood vessels as white edges. Furthermore, the t2 modality is a 2D-weighted image with 2–6 mm slice thicknesses; a t2-weighted flair modality produces 2D axial, coronal, or sagittal images with 2–6 mm slice thicknesses. Finally, a binary segmented image represents the tumor as the white component. Table 1 demonstrates the demographic information of 369 patients, which is used in this work. Upon successful execution of the pre-processing phase, the ETISTP model enters the tumor identification phase, which is detailed below.

### 3.2. Tumor Identification

**Figure 4:**
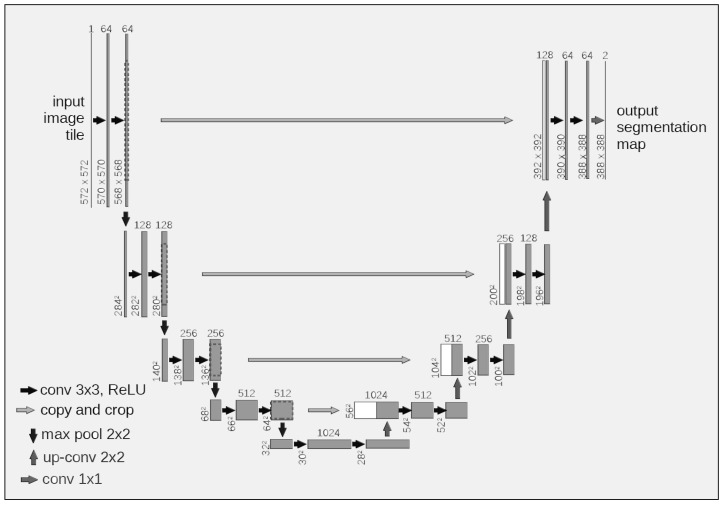
U-Net architecture for segmentation.

This section identifies the infected area in MRIs. Early and accurate identification is crucial for better cures, making this phase critical in the proposed ETISTP model. To achieve this, we employed image segmentation techniques to extract the tumor-infected areas from 3D MRIs. Various segmentation techniques are available in the literature [48], but we chose to use the Universal Network (U-Net) model [6] for semantic segmentation of the brain tumor. This model consists of a contracting path that captures the context and a symmetric expanding path that enables accurate localization. Figure 4 shows the architecture of the U-Net used in this work, where each gray box corresponds to a multi-channel feature map, and the number of channels is specified on top of each box. A white box represents the copied feature maps, and an arrow indicates the direction of an operation.

**Figure 5:**
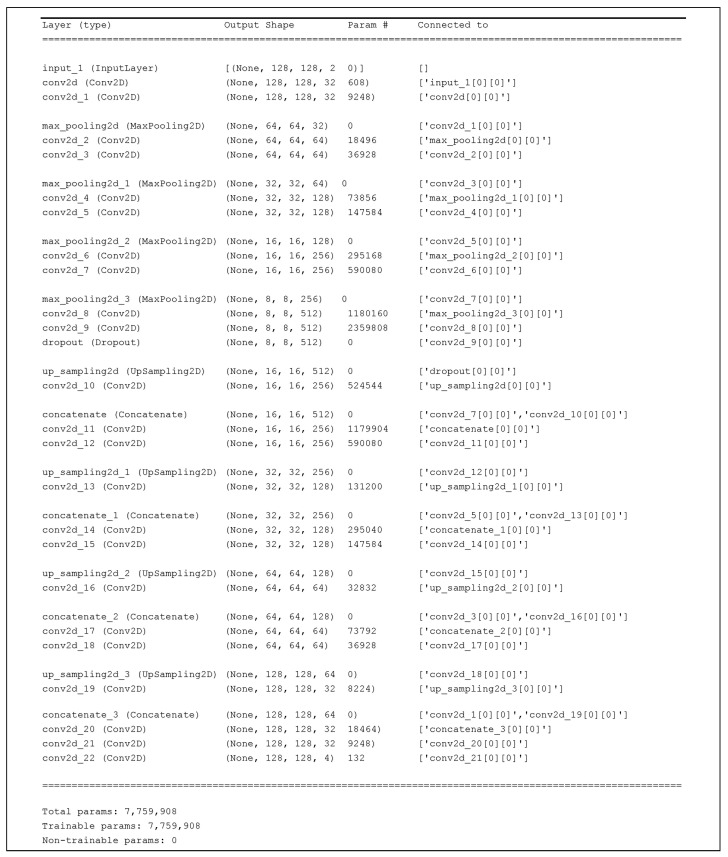
U-Net model summary.

**Figure 6:**
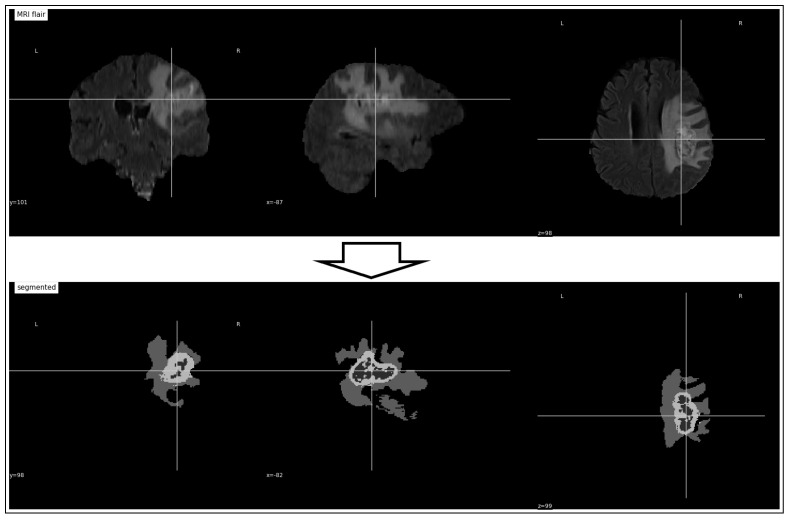
Example of the segmented tumor from the MRI.

The proposed ETISTP model trains the U-Net model using the aforementioned modalities to enhance brain tumor identification. Figure 5 depicts the U-Net training summary for tumor identification. The U-Net model produces a segmentation map from an input RGB image (height × width × 3) or a grayscale image (height × width × 1), with each pixel containing a class label represented as an integer. Figure 6 demonstrates a sample result in this regard. After training the U-Net model, it is validated on the same dataset. Some initial numerical results of tumor identification are shown in Table 2. Upon successful identification of the tumor, the ETISTP model initiates the next phase, which is detailed in the following subsection.

### 3.3. Tumor Volume Computation

**Figure 7:**
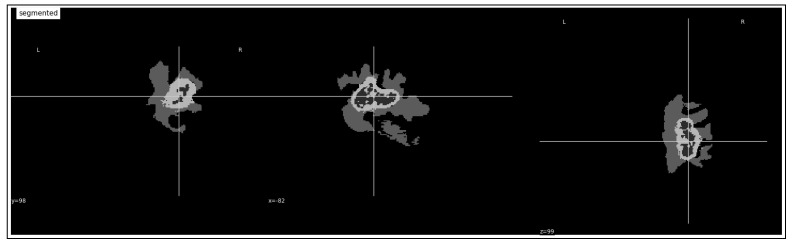
Example of a segmented 3D image in sagittal, coronal, and axial images.

This section focuses on the computation of the tumor volume. To the best of our knowledge, the ETISTP model is a pioneering approach that employs the tumor volume for the classification of tumors and the prediction of survival days using 3D MR images, as illustrated in Figure 7. A segmented image received from the previous phase is taken as the input. The volume of tumor (*V*) is computed using Equation (1) [49], as

(1) V=λμ,

where λ denotes the tumor area and μ refers to the height of the tumor in a 3D MR image, respectively. The tumor volume remains one of the inputs for survival time prediction, which is the final phase of our proposed ETISTP model.

### 3.4. Tumor Grade Classification

**Figure 8:**
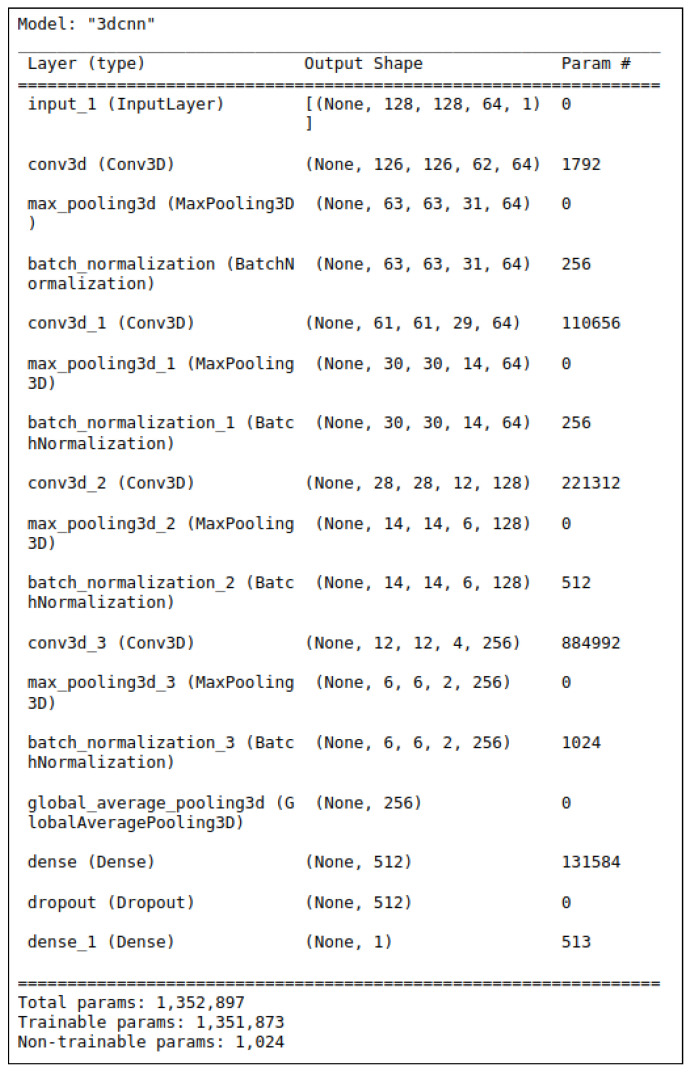
Classification of model parameters.

Glioma is a type of cancer that occurs in the brain and spinal cord; it originates from the gluey supportive cells. The ETISTP model classifies gliomas into LGG and HGG categories. LGGs are cancerous tumors that exhibit slow growth rates and arise from glial cells of the brain, whereas HGGs occur in the spinal cord and brain, have a fast growth rate, and are highly dangerous and difficult to treat. Classification involves grouping entities based on a set of ordered features. In this work, 3D convolutional neural networks (3D CNNs) [50,51,52] were employed to classify tumors into LGG and HGG categories. The 3D CNNs have successive convolution layers and rectified linear unit (ReLU) functions. Each layer consists of neurons that learn weights and biases from an input image and use weighted sums in the activation function. Hidden layers include conv3d, max_pooling3d, and batch_normalization, as shown in Figure 8. After training, the proposed model is tested, and initial results for the classification of tumors into HGG and LGG categories based on t1, t1ce, t2, flair, and segmented modalities are presented in Table 3.

The segmented image produced by the tumor identification phase is used as input for both the tumor volume computation and tumor grade classification phases. This allows the ETISTP model to perform both tasks simultaneously, minimizing the overall response time significantly. Upon completion of the tumor grade classification and tumor volume computation phases, the ETISTP model reaches the final phase, which is detailed below.

### 3.5. Survival Time Prediction

The diagnostic process involves identifying the brain tumor and its grade, which is the responsibility of the tumor identification and tumor grade classification phases of the proposed ETISTP model. However, this information alone is not sufficient to plan and enable better treatment. The survival time of a patient is crucial in this regard. Since medical practitioners cannot compute the exact survival time for a patient, the ETISTP model includes a prediction phase that estimates the number of days a patient may survive. This prediction will certainly assist medical practitioners in planning and implementing effective treatments against brain tumors.

In the literature, there are several prediction models; however, these models significantly lack accuracy. To this end, the ETISTP model integrates the patient’s age, EoGTR, tumor volume, and tumor grade. Tumor grades are indicated using binary values, i.e., zero (0) and one (1) for LGG and HGG, respectively. The ETISTP model employs the CoxPH model [34,36,53], which takes into account the tumor volume, tumor grade, age, and GTR status. This integration of different parameters helps to enhance the accuracy of survival time prediction, as will be demonstrated in Section 4.

The results of the Cox Proportional hazard (CoxPH) model provide data about the relationship between the four factors and the hazard ratio of survival time, as follows.

- The hazard ratio (HR) is the ratio of the hazard rates of two groups, which in this case is the ratio of the hazard rate of the group with a one-unit increase in the factor to the hazard rate of the group with the reference level of the factor.
- The coefficient (coef) of each factor is the estimated change in the log hazard ratio for a one-unit increase in the factor, holding other factors constant.
- The exponentiated coefficient (exp (coef)) is the estimated change in the hazard ratio for a one-unit increase in the factor, holding other factors constant.
- The standard error (se) of the coefficient is the estimated standard deviation of the coefficient.
- The 95% confidence interval (CI) of the coefficient provides a range of values for the coefficient that is likely to contain the true value of the coefficient with a 95% probability.
- The 95% CI of the exponentiated coefficient provides a range of values for the hazard ratio that is likely to contain the true value of the hazard ratio with a 95% probability.
- The z-value is the coefficient divided by the standard error and indicates the significance of the coefficient.
- The *p*-value is the probability of observing a z-value that is as extreme as (or more extreme than) the observed z-value under the assumption that the null hypothesis, which states that the coefficient is zero, is true.
- The −log2(p) is the negative logarithm (base 2) of the *p*-value and it indicates the strength of evidence against the null hypothesis.

Table 4 includes a summary of the parameters that are used to train the CoxPH with the aforementioned four parameters.

Table 5 demonstrates the coefficient results of the patients for each factor. These results can be interpreted as follows:

- Age: The coefficient of age is 0.04, indicating that the hazard ratio of survival time increases by 4% for a one-year increase in age, assuming that all other factors remain constant. This effect is statistically significant (z=5.45,p<0.005). The 95% confidence interval (CI) of the hazard ratio ranges from 1.02 to 1.05, indicating that the hazard ratio is likely to increase between 2% and 5% for a one-year increase in age.
- GTR: The coefficient of GTR is 0.04, which means that the hazard ratio of survival time increases by 4% for GTR, holding other factors constant. However, this effect is not statistically significant (z=0.52,p=0.60). The 95% CI of the hazard ratio is 0.90 to 1.19, which means that the hazard ratio can decrease by 10% or increase by 19% for GTR, but the uncertainty is high.
- Class: The coefficient of ’class’ is -0.64, which means that the hazard ratio of survival time decreases by 47% for class, holding other factors constant. This effect is marginally significant (z=−1.75,p=0.08), indicating weak evidence against the null hypothesis. The 95% CI of the hazard ratio is 0.26 to 1.08, which means that the hazard ratio can decrease by 74% or increase by 8% for class, but the uncertainty is high.
- Volume: The coefficient of volume is 0.00, which means that the hazard ratio of survival time does not change for volume, holding other factors constant. This effect is not statistically significant (z=−1.73,p=0.08). The 95% CI of the hazard ratio is 1.00 to 1.00, which means that the hazard ratio is likely to remain the same for volume. However, the upper bound of the CI is 1.08, indicating that there is a small possibility that the hazard ratio can increase by up to 8% for a one-unit increase in volume, but the uncertainty is high.

In summary, the CoxPH model indicates that age is a significant predictor of survival time, with a higher age associated with an increased hazard of death. However, the effects of GTR, class, and volume are less clear and require further investigation. It is important to note that the interpretation of these results should be made in the context of the study population and the specific research question being addressed.

Finally, the concordance of our trained CoxPH model is represented in Table 6. The concordance analysis result of the CoxPH model provides an assessment of the model’s predictive accuracy. In this case, the concordance value is 0.74, indicating that the model has a moderately good ability to discriminate between subjects who experience the event of interest (death) and those who do not. A perfect concordance value is 1.0, indicating perfect prediction accuracy, while a concordance value of 0.5 indicates random prediction. The partial AIC (Akaike information criterion) value is 1999.20, which is a measure of the model’s goodness-of-fit, with lower values indicating a better fit. The log-likelihood ratio test (LRT) value is 38.17 on 4 degrees of freedom (df), which compares the fit of the full model with a reduced model that does not include any of the predictor variables. The LRT assesses whether the addition of the predictor variables significantly improves the model’s fit, with a higher value indicating a better fit. In this case, the LRT value is significant, with a −log2(p) value of 23.20, indicating that the model with the predictor variables fits significantly better than the reduced model without any predictor variables. Overall, these results suggest that the CoxPH model with the four predictor variables (age, GTR, class, and volume) has a reasonably good ability to predict the survival time and provides a better fit than a model without any predictor variables.

We keep an 80% to 20% ratio for the training and testing in this phase. The ETISTP model computes the hazard values using Equation (2) [33,34], as

(2) $$h\left(t\right)=h_{o}\left(t\right)\sum_{i=1}^{n}exp\left(b_{i}w_{i}\right),$$

where *h* represents the hazard value, $h_{o}$ is a baseline hazard function that reflects the underlined hazard value for a case where all of the covariates are zero, *t* refers to the time at which a hazard value is recorded, *b* represents the hazard factor, *w* denotes the coefficient value for the concerned hazard value, and *n* (ranging from 0 through 3) refers to the risk factors.

Table 7 shows hazard factor weights for the risk factor, coefficient, and Exp (coef). In this model, the tumor volume has a coefficient of 0.01, but its hazard ratio is 1.00, which suggests that a one-unit increase in tumor volume does not significantly impact the risk of the event of interest. The tumor type has a coefficient of 0.15, and its corresponding hazard ratio is 1.16, indicating that patients with a different tumor type have a 16% higher risk of the event of interest, after adjusting for other variables in the model. The patient’s age has a coefficient of 0.03, and its corresponding hazard ratio is 1.04, indicating that for every one-year increase in age, the risk of the event of interest increases by 4% while controlling for other factors. The extent of GTR has a coefficient of 0.04, and its corresponding hazard ratio is 1.04, suggesting that patients with more extensive GTR have a 4% higher risk of the event of interest than those with less extensive GTR, after adjusting for other variables in the model. Overall, these results indicate that the tumor type, patient’s age, and the extent of GTR are significant predictors of the event of interest, while the tumor volume does not appear to have a significant effect on the risk of the event in this particular model.

ETISTP keeps the target value of the survival rate within the range of 0 to 1, where 0 refers to the minimum chances of the patient’s survival and 1 denotes the maximum chances. To compute the probability of a patient’s survival time in terms of days, the ETISTP model uses Equation (3) [53], as

(3) $$\ln S\left(t_{i}\right)=-\int_{0}^{t}h_{i}\left(t\right)d\left(t\right),$$

where *S* denotes the survival rate for a certain time *t* and *i* represent the time range, e.g., i=1,2,3,4,…,n. h(t) is the hazard value obtained from Equation (2) and d(t) is a derivative for the integration in the equation.

**Figure 9:**
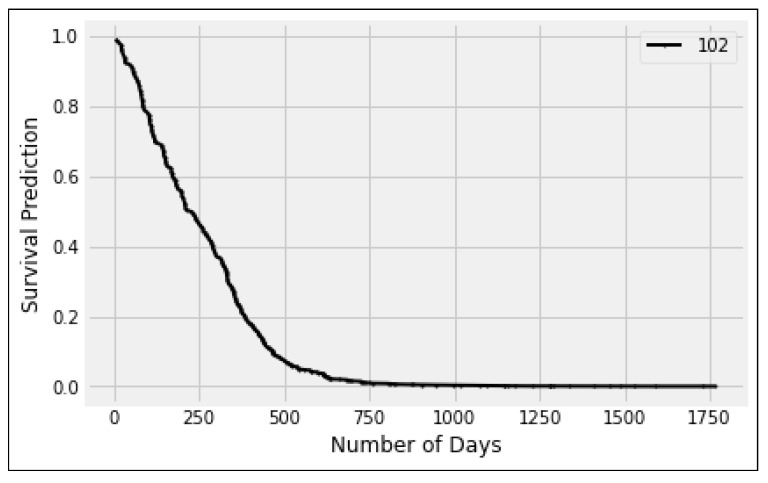
Single patient survival rate.

Figure 9 shows the initial results for the survival probability of a single patient with specifications provided in Table 8.

**Figure 10:**
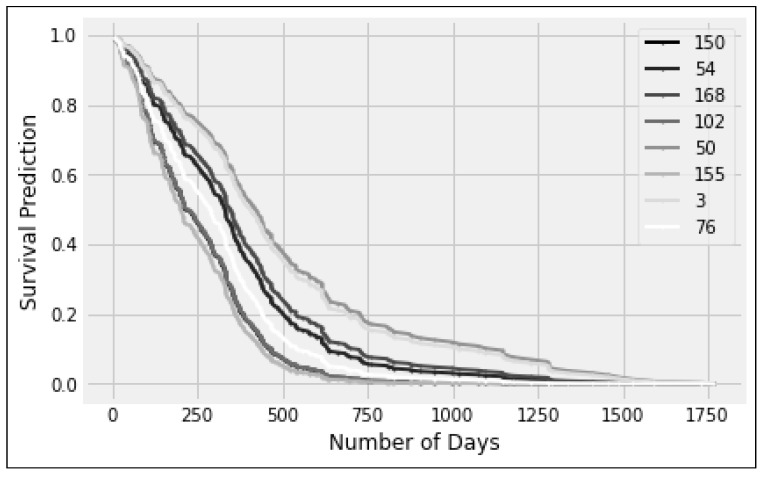
Survival rate of multiple patients.

**Figure 11:**
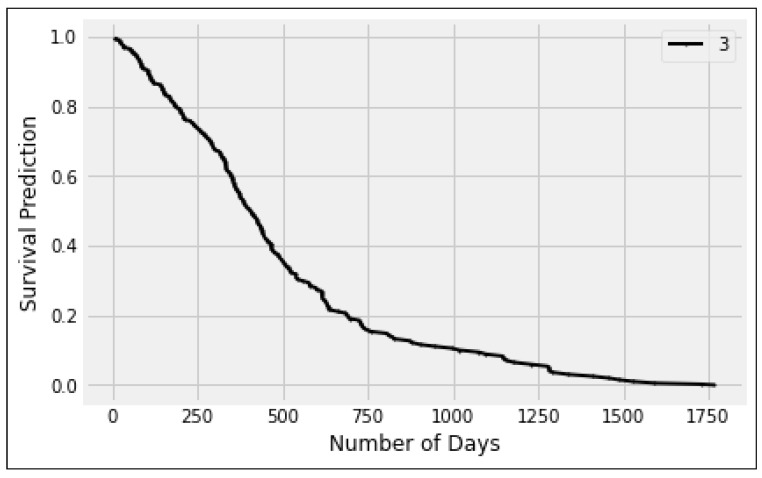
Survival rate for the LGG patient.

**Figure 12:**
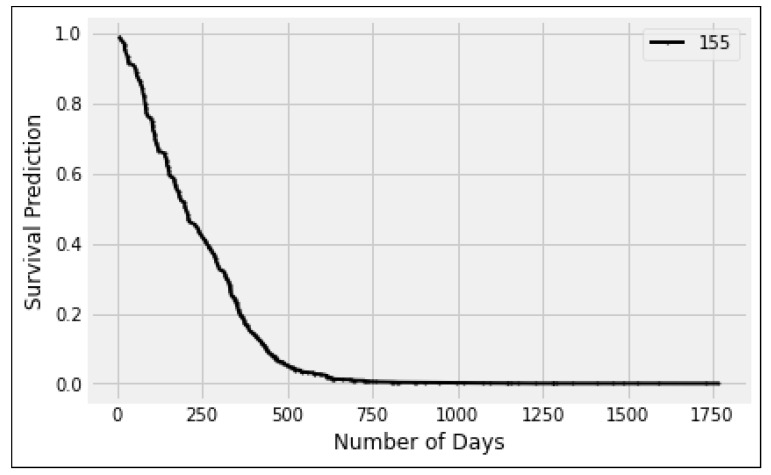
Survival rate for the HGG patient.

Similarly, the initial results for multiple patients are shown in Figure 10 with respect to the parameters specified in Table 9. Moreover, Figure 11 shows the survival rate for the LGG patient, whereas Figure 12 depicts the same for the HGG patient. These plots are based on the data provided in Table 10.

## 4. Performance Evaluation

This section evaluates the performance of our proposed ETISTP model. For tumor identification, the ETISTP model is compared with BU-Net [6], FCN [35], BrainSeg [54], and U-Net [24]. Moreover, to evaluate the classification performance, the proposed model is compared with 3DCNN [55] using five modalities, i.e., t1, t1ce, t2, Flair, and segmented. The survival prediction efficiency is evaluated in comparison with SAVAE-COX [39], CoxPH [40], PAGE-Net [41], the statistical machine learning algorithm [42], SVM [35], and the random forest classifier [20]. The following subsection details the simulation parameters used in this work.

### 4.1. Simulation Setup

The simulation results were obtained using TensorFlow v1.12 with Keras in an Anaconda environment, which provides extensive built-in library support. This was utilized for the state-of-the-art segmentation and classification of MRIs, brain tumor volume computation, and prediction of patients’ survival [56].

The hardware platform includes Dell OptiPlex 9020 with an Intel${}^{®}$ Core i$7^{\mathrm{TM}}$−4770 processor, 8 gigabytes of memory, and running Microsoft *©* Windows 10 (Home-20H2 Edition). Moreover, we used the Brats2020 dataset, which consists of MR images for 369 patients with 5 different modalities, i.e., t1, t1ce, t2, t2flair, and seg [10,22,44,45]. Each presented result is an average of over 20 replicated simulation runs, where all parameters were kept fixed, and only the input values were randomly changed.

### 4.2. Performance Evaluation Criteria

To evaluate the performance of the proposed ETISTP, we employed the Dice score for tumor identification [57,58]. Moreover, for the classification comparative analysis, this work used the confusion matrix for tumor classification [14,28,55]. Finally, the Concordance Index ($C_{i}$) was utilized to evaluate the performance of our proposed model in terms of survival prediction, which is widely used for survival analyses in different models [39,40].

### 4.3. Results and Discussion

This section presents the simulation results with their respective discussions. Figure 3 depicts sample MRI images in five modalities from the Brats2020 dataset.

#### 4.3.1. Tumor Identification

Tumor identification leads to effective survival prediction for a patient, which can enable healthcare centers to save the precious lives of patients. The Dice score is used to evaluate the tumor identification efficiency of the aforementioned models, which is used by state-of-the-art models for quantitative comparison. A Dice score identifies the similarity between two sets, e.g., *P* and *Q* [57], as

(4) $$Dice=\frac{2\times |P\cap Q|}{\left|P+Q\right|}$$

where |P| and |Q| refer to the cardinalities of the sets *P* and *Q*, respectively.

We compare our ETISTP model with the identification models presented in [6,20,24,35,54]. The results depicted in Table 11 show the superior performance of ETISTP, where it achieves a considerably high Dice score in comparison with the aforementioned models.

#### 4.3.2. Classification

The appropriate classification of brain tumors is crucial to enable effective cures and to predict a patient’s survival time. The ETISTP model classifies brain tumors in terms of LGG and HGG. To evaluate the classification performance, we employed confusion matrix, where accuracy, precision, and the F1 score, which determine the efficacy of a model. Classification accuracy is computed using Equation (5) [4], as

(5) $$Accuracy=\frac{S_{TP}+S_{TN}}{S_{TP}+S_{FP}+S_{TN}+S_{FN}},$$

where $S_{TP}$ and $S_{TN}$ denote true positive and true negative values, respectively. Similarly, $S_{FP}$ refers to the false positive value, whereas $S_{FN}$ is a false negative value. Furthermore, precision in classification is evaluated using Equation (6) [4], as

(6) $$Precision=\frac{S_{TP}}{S_{TP}+S_{FP}}.$$

Finally, the F1 score is calculated using Equation (7) [4], as

(7) $$F1score=\frac{S_{TP}}{S_{TP}+\frac{1}{2}\left(S_{FP}+S_{FN}\right)}.$$

**Figure 13:**
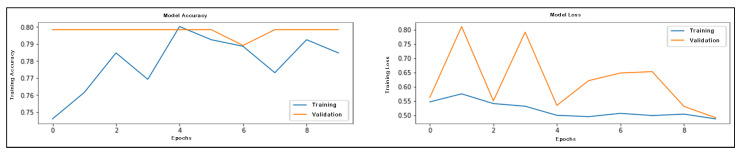
T1: Model accuracy and loss for 10 epochs.

**Figure 14:**
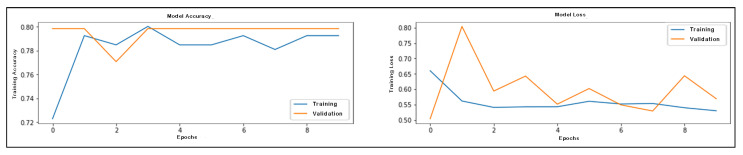
T1ce: Model accuracy and loss for 10 epochs.

**Figure 15:**
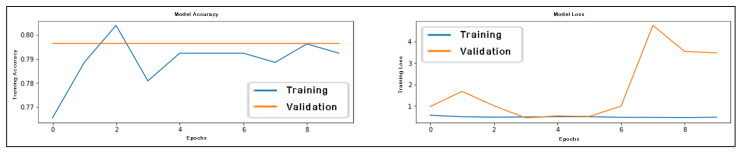
T2: Model accuracy and loss for 10 epochs.

**Figure 16:**
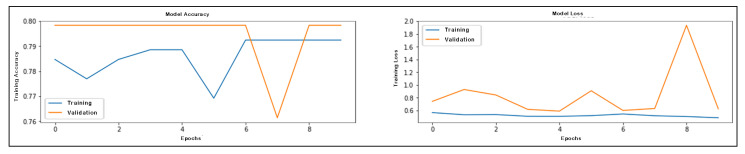
Flair: model accuracy and loss for 10 epochs.

**Figure 17:**
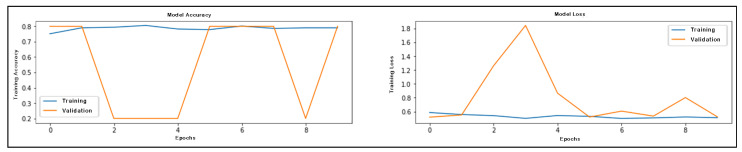
Segmented: model accuracy and loss for 10 epochs.

The classification efficacy of ETISTP, in terms of HGG and LGG, is evaluated in comparison with state-of-the-art models proposed in [4,14,28,55]. Table 12 presents the simulation results that affirm the superiority of the proposed ETISTP model in comparison with the aforementioned models. Moreover, the training accuracy and training loss for the five different modalities, i.e., t1 through seg, are shown in Figure 13, Figure 14, Figure 15, Figure 16 and Figure 17, respectively.

#### 4.3.3. Survival Time Prediction

This section presents simulation results obtained for the proposed ETISTP model in terms of survival time prediction, in comparison with existing models from the literature. Successful classification of brain tumors into LGG and HGG leads to the next step, i.e., survival time prediction. To this end, we use Harrell’s Concordance Index ($C_{i}$), which is widely used for survival analysis in various models [39,40]. The $C_{i}$ value ranges from 0 to 1, where $C_{i}$ < 0.5 indicates ineffective, whereas $C_{i}$ ≥ 0.5 indicates effective survival prediction. $C_{i}$ is evaluated using Equation (8), as

(8) $$C_{i}=\frac{\sum_{i,j}I\left(T_{i}>T_{j}\right)I\left(Y_{i}<Y_{j}\right)S_{j}}{\sum_{i,j}I\left(T_{i}>T_{j}\right)S_{j}}l,$$

where *i* and *j* denote a pair of subjects, $T_{i}$ and $T_{j}$ refer to the survival times, and $Y_{i}$ and $Y_{j}$ are the predicted risk scores, respectively. $S_{j}\in \left\{0,1\right\}$, where 0 indicates a subject as censored, where 1 shows otherwise. I(.) is the indicator function, which is interpreted as a fraction for subjects with correctly ordered risk scores. Here, $C_{i}$ remains proportional to the survival time in terms of days. To evaluate the ETISTP model for survival time prediction, we compare it with the models presented in [39,40,41,42]. The simulation results presented in Table 13 confirm our claim of enhanced performance, where the ETISTP model outperforms the aforementioned models by a considerable margin. Moreover, the survival rate is evaluated by the ETISTP model for brain tumor patients.

### 4.4. Computational Efficiency

This section evaluates the computational efficiency of our proposed ETISTP model in comparison with random forest (RF) [20], SVM [35], and CoxPH [34]. The execution time is taken in seconds (s) for each aforementioned model on its application to Brats2020. Since the ETISTP model enables the parallel execution of tumor volume computation and classification, it achieves the smallest execution time among the aforementioned models. To this end, Table 14 demonstrates the results that confirm our claim; the ETISTP *model outperforms* the rest by a significant margin.

## 5. Conclusions

Technology-assisted diagnosis plays a critical role in determining the efficacy of a healthcare system. Brain tumors are a major cause of death worldwide, and the survival time of patients is crucial for prioritizing treatment and improving outcomes. Gliomas are a particularly deadly type of brain tumor, and are further classified into low- and high-grade gliomas, which makes survival prediction even more challenging. The existing literature offers several survival prediction models that use different parameters, such as patient age, survival days, gross total resection status, tumor size, or tumor grade. However, a comprehensive model that integrates all of these parameters is still lacking. Moreover, these models use tumor sizes that adversely impact the accuracy of survival prediction. To this end, we propose a novel model, ETISTP, which integrates the patient’s age, survival days, gross total resection status, tumor grade, and brain tumor volume, to improve survival time prediction. The ETISTP model is a pioneering model that employs tumor volume to enhance the accuracy of survival prediction. Additionally, our model also minimizes computation time by enabling parallel execution of tumor volume computation and classification. The simulation results affirm our claim that the ETISTP model outperforms eminent survival prediction models from the literature in terms of survival time prediction. Future extensions of this work may consider the inclusion of artificial intelligence-based decision-making for treatment. Moreover, the proposed prediction model can further be validated using larger datasets. Additionally, different clinical variables, e.g., genetic information, can be investigated to further enhance the prediction process.

## Figures and Tables

**Table 1:** Demographic information of the patients in Brats-2020.

Factor	Minimum	Maximum
Age	18.975	86.652
Survival Days	5	1767
GTR Status	1 (N/A)	3 (GTR)
Tumor Volume	7.285	227.126
Tumor Class	76 (LGG)	293 (HGG)

**Table 2:** Summary of the tumor identification performance.

Dice Coef	Accuracy	Precision	Sensitivity	Specificity
0.25	0.96	0.95	0.90	0.99

**Table 3:** Performance of modality-wise classification.

Modality	Accuracy	Precision	F1 Score
T1	94.00	93.81	95.77
T1ce	94.00	93.81	95.77
T2	94.38	93.62	95.65
Flair	93.23	93.81	95.77
Segmented	94.38	93.81	95.77
**Average**	**94.20%**	**93.77%**	**95.75%**

**Table 4:** Summary of the parameters used for training the CoxPHFitter model.

Model	lifeline.CoxPHFitter
Duration Column	days
Event Column	event
Baseline Estimation	Breslow
Number of observations	228
Number of events observed	228
Partial log-likelihood	−994.44

**Table 5:** The parameter coefficient values from the *CoxPH* model.

Factor	Coef	Exp (Coef)	Se (Coef)	Coef Lower 95%	Coef Upper 95%	Exp (Coef) Lower 95%	Exp (Coef) Upper 95%	z	*p*	−log2(p)
Age	0.04	1.04	0.01	0.02	0.05	1.02	1.05	5.45	<0.005	24.26
GTR	0.04	1.04	0.07	−0.10	0.17	0.90	1.19	0.52	0.60	0.73
Class	−0.64	0.53	0.37	−1.36	0.08	0.26	1.08	−1.75	0.08	3.63
Volume	0.00	1.00	0.00	−0.00	0.08	1.00	1.00	−1.73	0.08	3.58

**Table 6:** Concordance results of the CoxPH model.

Concordance	0.74
Partial AIC	1999.20
Log-likelihood ratio test	38.17 on 4 df
−log2(p) of ll-ratio test	23.20

**Table 7:** Hazard Factor Weight.

Risk Factor	Coefficients	Exp (Coef)
Tumor Volume	0.01	1.00
Tumor Type	0.15	1.16
Patient’s Age	0.03	1.04
Extent of GTR	0.04	1.04

**Table 8:** Single Patient input data.

Patient’s No.	Age (Years)	GTR	Tumor Class	Volume
102	85.942	1	HGG	58.208

**Table 9:** Sample input data for survival prediction.

Patient’s No.	Age	GTR	Class	Volume
150	63.805	3	1	095.391
054	66.510	3	2	118.394
168	64.378	3	2	099.624
102	85.942	1	2	058.208
050	52.348	3	2	121.570
155	81.112	3	2	162.623
003	39.068	1	1	103.496
076	79.211	1	2	050.183

**Table 10:** Input data for LGG and HGG patients.

Patient’s No.	Age (Years)	GTR	Tumor Class	Volume
3	39.068	1	LGG	103.496
155	81.112	3	HGG	162.623

**Table 11:** Tumor identification results with respect to the Brats2020 dataset.

S No.	Author(s)	Dice Score
1.	Rehman et al. [54]	0.790
2.	Rehman et al. [6]	0.837
3.	Amian et al. [20]	0.840
4.	Ilhan et al. [24]	0.880
5.	Islam et al. [35]	0.899
**6.**	**The proposed ETISTP model**	**0.902**

**Table 12:** Classification of a brain tumor with respect to LGG and HGG.

S No.	Author(s)	Accuracy	Precision	F1 Score
1.	Chenjie et al. [28]	88.22%	86.76%	85.18%
2.	Zahraa et al. [55]	91.02%	87.07%	88.44%
3.	Attique et al. [14]	92.50%	88.37%	89.12%
4.	Narmatha et al. [4]	93.85%	94.77%	95.42%
**5.**	**The proposed ETISTP model**	**94.20%**	**95.77%**	**95.75%**

**Table 13:** Patient’s survival prediction based on the Brats2020 dataset.

S No.	Author(s)	Method(s)	Concordance	*p*-Value
1.	Moradmand et al. [40]	CoxPH	0.58	0.006
2.	Hao et al. [41]	PAGE-Net	0.64	0.007
3.	Senders et al. [42]	Statistical machine learning algorithm	0.69	0.008
4.	Meng et al. [39]	SAVAE-COX	0.71	0.009
**5.**	**The proposed ETISTP model**	CoxPH using the integrated parameters	**0.74**	**0.050**

**Table 14:** Computational efficiency analysis.

S No.	Model(s)	Time
1.	RF [20]	12.348 s
2.	SVM [35]	8.692 s
3.	CoxPH [34]	7.417 s
**4.**	**The proposed ETISTP model**	**0.005 s**

## Data Availability

Not applicable.
